# Virome and Blood Meal-Associated Host Responses in *Ixodes persulcatus* Naturally Fed on Patients

**DOI:** 10.3389/fmicb.2021.728996

**Published:** 2022-02-17

**Authors:** Liang-Jing Li, Nian-Zhi Ning, Yuan-Chun Zheng, Yan-Li Chu, Xiao-Ming Cui, Ming-Zhu Zhang, Wen-Bin Guo, Ran Wei, Hong-Bo Liu, Yi Sun, Jin-Ling Ye, Bao-Gui Jiang, Ting-Ting Yuan, Jie Li, Cai Bian, Lesley Bell-Sakyi, Hui Wang, Jia-Fu Jiang, Ju-Liang Song, Wu-Chun Cao, Tommy Tsan-Yuk Lam, Xue-Bing Ni, Na Jia

**Affiliations:** ^1^State Key Laboratory of Pathogen and Biosecurity, Beijing Institute of Microbiology and Epidemiology, Beijing, China; ^2^Mudanjiang Forestry Central Hospital, Mudanjiang, China; ^3^The Affiliated Hospital of Shandong University of Traditional Chinese Medicine, Jinan, China; ^4^Chinese PLA Center for Disease Control and Prevention, Beijing, China; ^5^Shanghai Institute for Emerging and Re-emerging Infectious Diseases, Shanghai Public Health Clinical Center, Shanghai, China; ^6^Department of Infection Biology and Microbiomes, Institute of Infection, Veterinary, and Ecological Sciences, University of Liverpool, Liverpool, United Kingdom; ^7^State Key Laboratory of Emerging Infectious Diseases and Centre of Influenza Research, School of Public Health, The University of Hong Kong, Pok Fu Lam, Hong Kong SAR, China; ^8^Joint Institute of Virology (SU/HKU), Shantou University, Shantou, China

**Keywords:** ticks, bloodmeal, virome, reactive oxygen species, patients, Jingmen tick virus

## Abstract

The long-lasting co-evolution of ticks with pathogens results in mutual adaptation. Blood-feeding is one of the critical physiological behaviors that have been associated with the tick microbiome; however, most knowledge was gained through the study of laboratory-reared ticks. Here we detached *Ixodes persulcatus* ticks at different stages of blood-feeding from human patients and performed high-throughput transcriptomic analysis on them to identify their virome and genes differentially expressed between flat and fully fed ticks. We also traced bloodmeal sources of those ticks and identified bats and three other potential mammalian hosts, highlighting the public health significance. We found Jingmen tick virus and 13 putative new viruses belonging to 11 viral families, three of which even exhibited high genetic divergence from viruses previously reported in the same tick species from the same geographic region. Furthermore, differential expression analysis suggested a downregulation of antioxidant genes in the fully fed *I. persulcatus* ticks, which might be related to bloodmeal-related redox homeostasis. Our work highlights the significance of active surveillance of tick viromes and suggests a role of reactive oxygen species (ROS) in modulating changes in the microbiome during blood-feeding.

## Introduction

Zoonotic pathogens, which reside in animal reservoir species and may spill over into the human population, are emerging at an unprecedented rate (Jones et al., 2008; Shi et al., 2016). Vectors exacerbate and complicate zoonotic disease transmission. Ticks are obligate blood-feeding vectors that parasitize a wide range of animals, including reptiles, birds, and mammals. As a result of their feeding on blood, these arthropods are versatile vectors transmitting a plethora of pathogenic agents, including viruses, bacteria, helminths, and protozoa (de la Fuente et al., 2008; Jia et al., 2020). Blood-feeding is a key physiological process providing essential nutrients for ticks, mainly dietary hemoglobin which is acquired as an exogenous source of heme and thereby sequestered by vitellins in the developing oocytes (Perner et al., 2016). The blood-feeding strategy of ticks provides an ideal system for pathogen transmission, benefiting from their multiple bloodmeals on different hosts to accomplish their complete life cycle and their relatively long-lasting attachment to hosts for several days or weeks (Nava et al., 2009).

Screening of a fed tick detached from a patient is important to evaluate the risk to human health; however, most questions related to this natural, field-collected feeding tick are unsolved. Firstly, identification of the host species on which the tick fed during its previous parasitic stage is imperative to predict the risk of pathogen transmission; however, there are only limited reports of tracing the host species of tick blood meals (Allan et al., 2010; Önder et al., 2013). Secondly, the vector-borne virome has been increasingly highlighted in identifying new pathogens; however, the virome is only available for about 10 tick species (Tokarz et al., 2014, 2018; Pettersson et al., 2017; Harvey et al., 2019; Meng et al., 2019; Vandegrift and Kapoor, 2019) among over 800 tick species worldwide (Jongejan and Uilenberg, 2004). Thirdly, studies have demonstrated that the host bloodmeal can significantly influence the tick microbiome in various ways, which may fundamentally impact the transmission of zoonotic pathogens in these natural systems (Hawlena et al., 2013; Zhang et al., 2014; Rynkiewicz et al., 2015; Swei and Kwan, 2017; Charrier et al., 2018; Zolnik et al., 2018). However, most blood-feeding ticks in previous studies were reared under laboratory conditions, whereas field-collected ticks were rarely analyzed.

To fill the knowledge gaps mentioned above, we detached *Ixodes persulcatus* at different blood-feeding stages from patients and carried out a transcriptomic analysis. The hard tick *I. persulcatus* is widely distributed in Northern China and is most commonly responsible for tick-borne diseases in humans in this region (Cao et al., 2000, 2003). *Ixodes persulcatus* transmits a various disease-causing bacteria, including genera *Borrelia*, *Rickettsia*, *Anaplasma*, *Francisella*, *Coxiella*, and *Ehrlichi*a. We aimed to identify the possible animal hosts on which the ticks had fed during the previous parasitic stage, elucidate the virome of detached ticks using transcriptomic approaches, and explore blood meal-related tick responses. Our study may help to identify new biotic drivers indicating new strategies to control ticks and the pathogens they transmit.

## Materials and Methods

### Tick Collection

Ticks feeding on patients who sought treatment at Mudanjiang Forestry Central Hospital in Heilongjiang Province, China, were collected between May and August 2016. The study was approved by the ethics committee of the hospital (Mudanjiang Forest Central Hospital 2011-03) in accordance with the medical research regulations of China. The species and developmental stage were identified by an entomologist (Yi Sun). Adult *I. persulcatus* ticks were included in the study, and the species was later confirmed by analyzing sequences of the mitochondrial cytochrome *c* oxidase subunit I gene. We further divided the feeding ticks into three groups: flat (ticks that had imbibed small amounts of blood and had not started to enlarge), partially fed (ticks that had begun to enlarge but were not fully engorged), and fully fed (ticks that had fully engorged and were almost ready to detach). All samples were captured alive and thoroughly surface-sterilized (two successive washes in 70% ethanol for 30 s each) before storage at −80°C for DNA/RNA extraction.

### Sample Preparation and Sequencing

Extraction of total DNA and RNA from ticks was performed using the AllPrep DNA/RNA Mini Kit (Qiagen, Valencia, CA, United States) with modifications. Briefly, ticks were homogenized in RLT solution under liquid nitrogen. The homogenate was then incubated at 55°C for 10 min with proteinase K (Qiagen) and centrifuged for 30 s at 15,000 × *g*. The homogenized lysate was transferred to an AllPrep DNA spin column and centrifuged for 30 s at 8,000 × *g*. The AllPrep DNA spin column was used for later DNA purification, and the flow-through was used for RNA purification as per the manufacturer’s instructions. Ten ticks were pooled together to extract RNA/DNA, and each group comprised three pools.

The extracted RNA was used for transcriptome sequencing (RNA-seq) after RNA quantification and quality checking. The rRNA was removed using RiBo-Zero Gold rRNA removal reagents (human/mouse/rat) (Illumina). Then, the sequencing library was prepared following the Illumina standard protocol. Paired-end (2 × 150 bp) sequencing of the RNA library was performed on an Illumina HiSeq 4000 platform at Novogene Tech (Beijing, China).

### Discovery and Assembly of Viral Genomes

RNA sequencing reads were *de novo* assembled using the Trinity program (Grabherr et al., 2011). Assembled contigs were subject to BLASTx against all non-redundant (nr) database downloaded from GenBank, and the threshold *E* value was set to 1e-5. Putative viral contigs were further merged by high-identity overlaps using the SeqMan program of Lasergene package v7.1 (DNAstar, Madison, WI, United States). Original reads were aligned to the contigs again using Bowtie2 (Langmead and Salzberg, 2012) to complete the remaining gaps, and the assembly was verified in the Integrated Genomics Viewer (Thorvaldsdóttir et al., 2013).

### Phylogenetic Analyses

The highly conserved RNA-dependent RNA polymerase (RdRp) gene was used to construct family-level phylogenies comprising the viruses that were highly divergent. Predicted viral proteins of the RdRp genes in this study were aligned with reference proteins of the same viral families using the E-INS-i algorithm in MAFFT version 7 (Katoh and Standley, 2013). Ambiguously aligned regions were removed using TrimAl (Capella-Gutiérrez et al., 2009). The WAG + Γ model was identified as the best-fit amino acid substitution model using Prot-Test 3.4 (Darriba et al., 2011), which was used for maximum likelihood (ML) phylogeny reconstruction with bootstrap tests (1,000 replicates) in PhyML version 3.0 (Guindon and Gascuel, 2003). The ML trees were visualized with FigTree v1.4.2.

### Differential Expression Analysis

A single *de novo* assembly across all samples was generated using Trinity (Grabherr et al., 2011), then reads from each sample were separately aligned back to the single Trinity assembly for downstream analyses of the differential expression of transcripts. Transcript abundance containing RNA-Seq fragment counts for each transcript (or gene) across each sample was estimated using the alignment-based abundance estimation method RSEM (Li et al., 2009). The trimmed mean of M-values normalization method (TMM) were subsequently used to normalize the transcript abundance. Differentially expressed (DE) transcripts or genes were identified by Fisher’s oexact test running “*edge*” R packages (Robinson et al., 2010; McCarthy et al., 2012), and then those transcripts or genes that had *p*-values of at most 5e-2 and were at least 2^1.5-fold differentially expressed were extracted to generate a sample correlation matrix heatmap and a DE gene vs. samples heatmap using Trinity downstream analysis script. The DE genes shown in the heatmap were grouped into gene clusters with similar expression patterns by cutting the hierarchically clustered gene tree at 60% from the tip. The functional annotations of the DE transcripts or genes were performed using Trinotate (Bryant et al., 2017) based on Pfam database. We classified these annotated transcripts or genes based on the COG database using *eggnog* mapper (Huerta-Cepas et al., 2017).

## Results

### Identification of Bloodmeal Source in Adult *Ixodes persulcatus*

**FIGURE 1:**
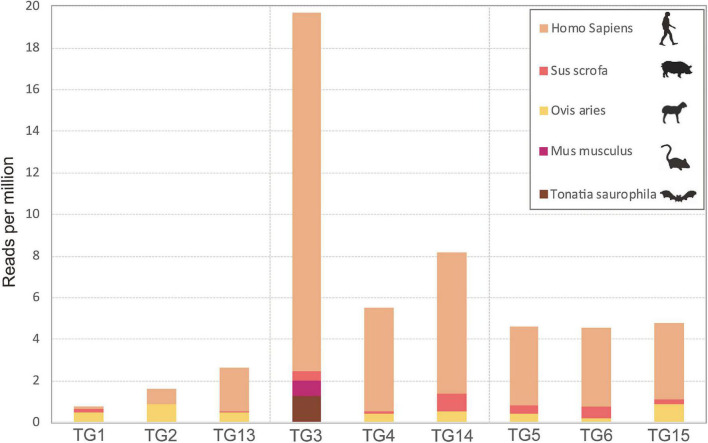
Expression levels of the cytochrome *c* oxidase subunit I gene of various host species revealed by RNAseq analysis of pools of *Ixodes persulcatus* ticks removed from human patients at Mudanjiang Forestry Central Hospital in Heilongjiang Province, China. Ticks were divided into three groups each comprising three pools of ten ticks: flat (pools TG1, TG2, and TG13), partially fed (pools TG3, TG4, and TG14), and fully fed (pools TG5, TG6, and TG15).

A total of 90 feeding adult *I. persulcatus* ticks were collected from patients. We sequenced nine pools of ticks (10 ticks/pool) by transcriptomic survey. Pools TG1, TG2, and TG13 comprised flat ticks; pools TG3, TG4, and TG14 comprised partially fed ticks; and pools TG5, TG6, and TG15 comprised fully fed ticks. We analyzed *cox1* genes from RNA-seq data to identify the source of previous bloodmeals of *I. persulcatus* and found five mammal host sources, namely, *Tonatia saurophila*, *Mus musculus, Ovis aries*, *Sus scrofa*, and *Homo sapiens* (Figure 1). The host *cox1* gene sequences assembled in the study had over 98.5% identity to the respective reference sequences (Supplementary Table 1). The most abundant *cox1* sequence was from *Homo sapiens*, accounting for 1,570 reads across all samples. Intriguingly, one bat species, *Tonatia saurophila*, was detected with a read count of 48 in one of the partially fed tick pools (Figure 1 and Supplementary Table 1).

### *Ixodes persulcatus* Tick Virome

**FIGURE 2:**
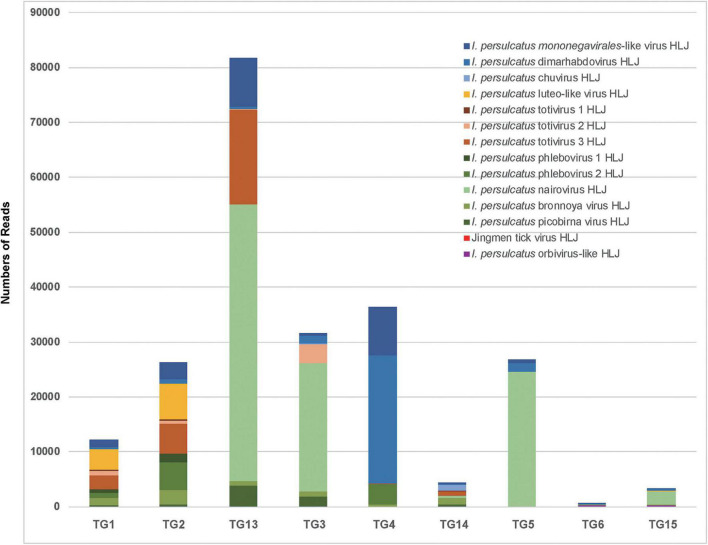
Detection by RNAseq of known and novel viruses in pools of *I. persulcatus* ticks removed from human patients at Mudanjiang Forestry Central Hospital in Heilongjiang Province, China. Levels of abundance of Jingmen tick virus and 13 newly discovered viruses in flat (pools TG1, TG2, and TG13), partially fed (pools TG3, TG4, and TG14), and fully fed (pools TG5, TG6, and TG15) ticks.

We performed nine RNA-seq runs on each of the nine pools of *I. persulcatus* ticks, generating 72 GB of total sequence data. Among the nine resultant RNA-seq libraries, library TG13 (flat ticks) had the most abundant viral reads, followed by library TG4 (partially fed ticks), while libraries TG6 and TG15 (fully fed ticks) had the fewest viral reads (Figure 2). Through a BLASTx search with the assembled genome sequences, we discovered 14 known or novel viruses belonging to 11 viral families (Table 1). All of which had fully or almost fully intact genomes with undisrupted reading frames. These included, with proposed names of new viruses in brackets, one *Mononegavirales*-like virus (*I. persulcatus mononegavirales*-like virus HLJ), one novel chuvirus (*I. persulcatus* chuvirus HLJ), one novel rhabdovirus (*I. persulcatus* dimarhabdovirus HLJ), three distant relatives of *Totiviridae* (*I. persulcatus* totivirus 1 HLJ, *I. persulcatus* totivirus 2 HLJ and *I. persulcatus* totivirus 3 HLJ), one novel *Partitiviridae* (*I. persulcatus* picobirna virus HLJ), one novel orbivirus (*I. persulcatus* orbivirus-like HLJ), one *Luteoviridae*-like virus (*I. persulcatus* luteo-like virus HLJ), four distant relatives of *Bunyavirales* (*I. persulcatus* phlebovirus 1 HLJ, *I. persulcatus* phlebovirus 2 HLJ, *I. persulcatus* nairovirus HLJ, *I. persulcatus* bronnoya virus HLJ), and Jingmen tick virus (JMTV). Reads from *I. persulcatus* nairovirus HLJ were most abundant, followed by reads from *I. persulcatus* dimarhabdovirus HLJ (Figure 2). These viruses shared 36–99% amino acid identities compared with previously reported viruses, and the query coverage varied from around 30 to 90% (Table 1).

**TABLE 1:** Summary of the viruses discovered by RNAseq in *Ixodes persulcatus* ticks removed from human patients at Mudanjiang Forestry Central Hospital in Heilongjiang Province, China.

Proposed name	Length	Closest Blastx hit	Identity %	Query Coverage %	Abundance (reads count)
*Ixodes persulcatus* mononegavirales-like virus HLJ	10,865	Deer tick mononegavirales-like virus RNA-dependent RNA polymerase gene	97.64	36	23,431
*Ixodes persulcatus* phlebovirus 1 HLJ	6,696	Blacklegged tick phlebovirus 3 L gene	74.66	97	2,156
*Ixodes persulcatus* phlebovirus 2 HLJ	6,715	Norway phlebovirus 1 L gene	87.28	98	9,687
*Ixodes persulcatus* picobirna virus	1,639	Norway partiti-like virus 1 RNA-dependent RNA-polymerase	93.82	91	6,825
*Ixodes persulcatus* totivirus 3 HLJ	5,615	Hubei toti-like virus 24 hypothetical protein 2	36.97	42	26,215
*Ixodes persulcatus* bronnoya virus HLJ	9,320	Bronnoya virus RNA-dependent RNA-polymerase	38.85	88	7,756
*Ixodes persulcatus* totivirus 1 HLJ	2,682	Lonestar tick totivirus polymerase	39.8	98	672
Ixodes persulcatus luteo-like virus HLJ	2,646	Norway luteo-like virus 2 RNA-dependent RNA-polymerase	93.6	89	10,215
*Ixodes persulcatus* dimarhabdovirus HLJ	10,365	Norway mononegavirus 1 RNA-dependent RNA-polymerase	49.46	61	28,533
*Ixodes persulcatus* totivirus 2 HLJ	8,914	Lonestar tick totivirus polymerase	39.96	31	4,857
*Ixodes persulcatus* nairovirus HLJ	14,779	Beiji nairovirus RNA-dependent RNA polymerase	99.43	85	101,149
*Ixodes persulcatus* orbivirus-like HLJ	4,017	Skunk River virus VP1 gene	40.26	94	560
Jingmen tick virus HLJ	2,313	Jingmen tick virus NS5-like protein gene	92.6	99	84
*Ixodes persulcatus* chuvirus HLJ	11,440	Blacklegged tick chuvirus 2 L gene	83.07	57	1,229

**FIGURE 3:**
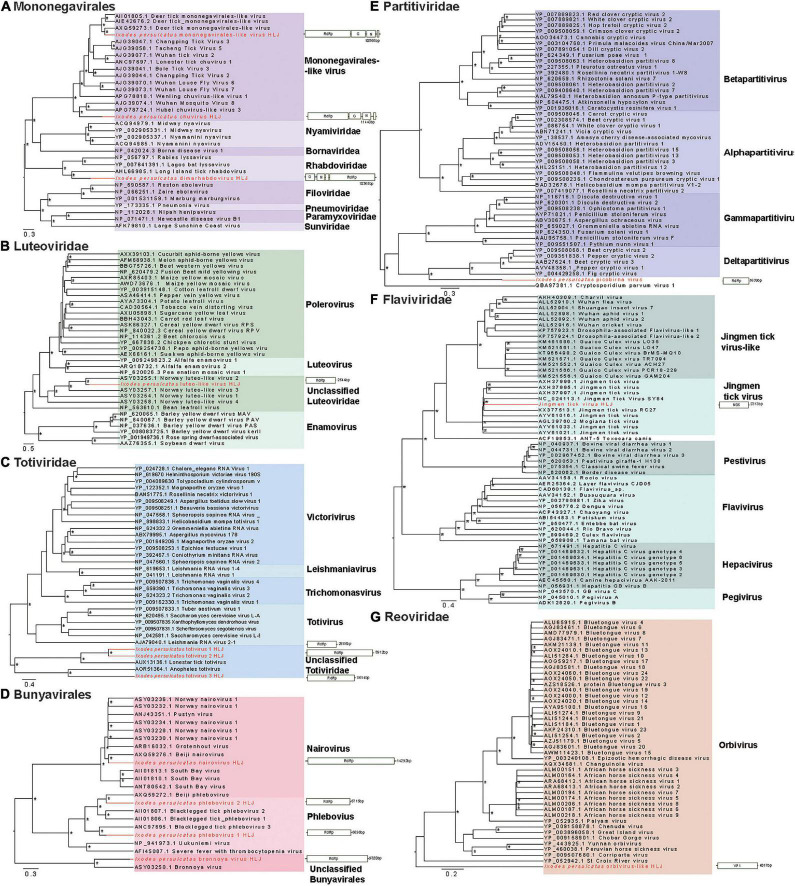
Phylogenetic trees of the RNA-dependent RNA polymerase (RdRp) gene-based on representative amino acid sequences and schematic genome structures of 14 viruses detected in *I. persulcatus* ticks removed from human patients at Mudanjiang Forestry Central Hospital in Heilongjiang Province, China. (A) *Mononegavirales*; (B) *Luteoviridae*; (C) *Totiviridae*; (D) *Bunyavirales*; (E) *Partitiviridae*; (F) *Flaviviridae*; and (G) *Reoviridae*. Support values above 0.7 are indicated by asterisk. The trees were mid-point rooted.

We identified three viruses in the *Mononegavirales*. Among them, *I. persulcatus mononegavirales*-like virus HLJ was closely related to deer tick *mononegavirales*-like virus, which had been previously reported in Heilongjiang Province, China (Meng et al., 2019; Figure 3A). *Ixodes persulcatus* dimarhabdovirus HLJ clustered with Long Island tick rhabdovirus and formed a monophyletic clade with other rhabdoviruses (Figure 3A). *Ixodes persulcatus* chuvirus HLJ had 83% amino acid similarity with blacklegged tick chuvirus-2 but only 57% coverage of the L segment sequence. We obtained the nearly complete genomes of these three viruses. The genome of *I. persulcatus mononegavirales*-like virus HLJ, *I. persulcatus* dimarhabdovirus HLJ, and *I. persulcatus* chuvirus HLJ were 10,865, 10,365, and 11,440 nucleotides (nt) long, and all encoded four predicted ORFs.

Three distant relatives of *Totiviridae* (*I. persulcatus* totivirus 1 HLJ, *I. persulcatus* totivirus 2 HLJ, *I. persulcatus* totivirus 3 HLJ) had extremely low amino acid identity (37–40%) with previously described viruses. Their RdRp sequences were highly divergent from the existing clades and fell into the unclassified groups on the phylogenetic tree (Figure 3C). This indicates the distinct phylogenetic position of these newly identified viruses from the currently known viruses.

Three members of the *Bunyavirales*, *I. persulcatus* phlebovirus 1 HLJ, *I. persulcatus* phlebovirus 2 HLJ, and *I. persulcatus* nairovirus HLJ showed high amino acid identities of 75, 87, and 99% with previously reported blacklegged tick phlebovirus 3, Norway phlebovirus 1, and Beiji nairovirus, respectively, based on the RdRp gene. The fourth member of the *Bunyavirales*, *I. persulcatus* bronnoya virus HLJ exhibited 38% amino acid identity with Bronnoya virus (88% sequence coverage of RdRp gene). The phylogenetic tree of *Bunyavirales* indicated that the two phleboviruses and the nairovirus clustered with previously reported Chinese strains and formed well-supported monophyletic groups closely related to the “classic” phleboviruses and nairoviruses (Figure 3D). It is suggested that they may share a single common ancestor for this gene. The newly discovered Bronnoya virus RdRp sequence clustered with the previously reported Bronnoya virus.

*Ixodes persulcatus* luteo-like virus HLJ, *I. persulcatus* picobirna virus HLJ, and JMTV found in this study exhibited 87, 94, and 99% amino acid identities to previously reported viruses with, respectively, 98, 91, and 93% sequence coverage (Figures 3B,E,F).

*Ixodes persulcatus* orbivirus-like HLJ revealed 40% amino acid similarity across 94% of the VP1 gene sequence of the orbivirus Skunk River virus (MK100569.1), and it showed a phylogenetic position distinct from other orbiviruses in the phylogenetic tree (Figure 3G).

### Bloodmeal-Associated Differential Gene Expression Analyses

**FIGURE 4:**
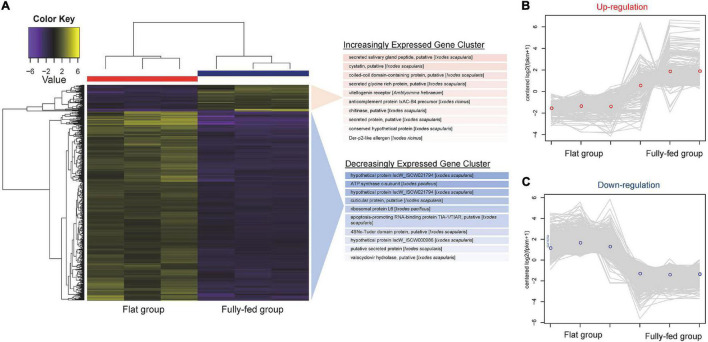
RNAseq analysis of tick gene transcripts detected in pools of flat and fully fed *I. persulcatus* ticks removed from human patients at Mudanjiang Forestry Central Hospital in Heilongjiang Province, China. (A) Clustered heatmap of genes differentially expressed (DE) between flat and fully fed ticks. The top 10 most significant genes in the increasing and decreasing expression clusters are shown. The expression patterns of DE genes in each tick pool are shown for (B) upregulated genes and (C) downregulated genes.

We next detected the differential expression responses of ticks at two different stages of feeding: flat (pools TG1, TG2, and TG13) and fully fed (pools TG5, TG6, and TG15) to investigate the effect of the bloodmeal. We first generated a single Trinity assembly across all pools and then calculated the abundance separately for each pool. The subsequent differential expression analysis showed that a total of 1,302 genes were differently expressed (*p* < 0.05, logFC > 1.5). Most of the DE genes were negatively correlated (Supplementary Figure 1). As shown in Figure 4A, most of the DE genes (71.4%) were downregulated in fully fed ticks; of these, ATP synthase c-subunit, a putative cuticular protein, ribosomal protein L6, and the putative apoptosis-promoting RNA-binding protein TIA-1/TIAR were the most downregulated (Supplementary Table 2). The gene encoding a putative secreted salivary gland peptide was most upregulated at engorgement, followed by a putative cystatin, putative coiled-coil domain-containing protein, and putative secreted glycine-rich protein (Supplementary Table 2). One antimicrobial peptide, lysozyme, was downregulated in fully fed ticks (Supplementary Table 3).

All DE genes shown in the heatmap (Figure 4A) were grouped into clusters of genes with similar expression patterns. A total of 251 transcripts were clustered in the upregulation category (Figure 4B), while 930 transcripts were clustered in the downregulation category (Figure 4C). According to the taxonomy classification based on the COG database, 18.7% of DE genes in the upregulated category were related to post-translational modification, protein turnover, and chaperones, and 12.7% of upregulated DE genes displayed functions of translation, ribosomal structure, and biogenesis (Supplementary Figure 2A). Most of the DE genes in the downregulated category were related to functions of nucleotide transport and metabolism (18.2%) and cytoskeleton (18.3%) (Supplementary Figure 2B).

### Reactive Oxygen Species

Reactive Oxygen Species (ROS) produced by the mosquito midgut play a role in maintenance of redox homeostasis (Citelli et al., 2007; Hernandez et al., 2019), and previous literatures also suggested that the balance between positive and negative aspects of ROS in ticks should be carefully maintained (Hernandez et al., 2019). To achieve redox homeostasis, the ticks have a complex anti-oxidant system composed of enzymes such as glutathione peroxidase, glutathione-*S* transferase, and so on (Hernandez et al., 2018, 2020; Kusakisako et al., 2018, 2020). We found that antioxidant genes including glutathione peroxidase, glutathione *S*-transferase, peroxidase, peroxiredoxin, and protein kinase C were all downregulated in the fully fed ticks compared with the flat ticks (Supplementary Table 3).

## Discussion

Blood-feeding is an essential behavior for ticks, and its role is far beyond that of providing nutrition and energy for molting, development, and vitellogenesis. Its requirement for transmission of tick-borne pathogens is another important function. The present study is the first report on the virome of ticks fed on patients who sought medical attention in a sentinel hospital in northeastern China. Using unbiased high-throughput sequencing platforms, we found that five mammalian species had served as hosts for *I. persulcatus*, including a bat. We also identified Jingmen tick virus and 13 new viruses belonging to 11 viral families. A downregulation of antioxidant genes associated with blood intake was observed in the fed ticks, which might be correlated with maintenance of the redox homeostasis.

It is important to identify the sources of *I. persulcatus* bloodmeals to aid in assessing the risk to patients of tick-borne disease transmission. Bats are some of the most notorious natural reservoir/hosts for many fatal viruses, such as Ebola virus, SARS virus, and SARS-CoV-2 virus (Calisher et al., 2006; Zhou et al., 2020). We found that some *I. persulcatus* ticks feeding on humans may have previously imbibed bat blood, highlighting the need for alertness on this tick species for its role in transmitting novel pathogens from bats to humans. Various host *cox1* genes were identified in our data, namely mouse, bat, sheep, wild boar, and human, indicating the high risk of interspecies transmission of tick-borne pathogens. Previous studies have developed DNA barcoding (Kent, 2009; Allan et al., 2010; Gariepy et al., 2012) or proteome profiling (Önder et al., 2013) techniques to identify sources of tick blood meals. However, the host identification success was limited due to the heavily degraded remnants of blood. We used the RNA-seq platform to identify the origin of the host bloodmeals of the pooled samples; however, the sensitivity and specificity of this approach should be further evaluated.

We found 14 viruses, belonging to 11 viral families, and most of them showed quite low similarity and query coverage compared with previously found viruses. Meng et al. found five species of viruses, including JMTV, in the same geographic area (Pettersson et al., 2017). We have previously reported that JMTV was pathogenic to humans (Jia et al., 2019), and the known range of this virus has rapidly expanded to cover Africa, South America, the Caribbean, and Europe (Temmam et al., 2019). We will extend our vigilance to other new viruses found in this study for their potential pathogenicity in humans, as these viruses were found in ticks directly detached from human patients. However, whether *I. persulcatus* is the vector of these viruses remains to be explored. In addition, the possibility of the new viruses being endogenous virus elements (Bell-Sakyi and Attoui, 2016) is very low because all the viruses were found to have fully or almost fully intact genomes, without disrupted reading frames.

Reactive oxygen species level seems to be a typical association with the maintenance of redox homeostasis in hematophagous arthropods, such as ticks (Lara et al., 2005; Citelli et al., 2007) and mosquitoes (Cirimotich et al., 2011; Oliveira et al., 2011). However, ticks might have developed different mechanisms to control ROS level compared with mosquitoes. For example, ticks have a different pathway for heme biosynthesis and require the acquisition of exogenous heme (Perner et al., 2016). Due to their feeding physiology, ticks are exposed to elevated amount of ROS. The ingestion of host blood containing pro-oxidant molecules, such as heme, could catalyze the production of ROS (Graça-Souza et al., 2006). Aside from blood-feeding, many cellular processes and enzyme activity could result in ROS production and eventually increase oxidative stress levels (Hernandez et al., 2019). Recently, carbohydrate metabolic compensation and peroxiredoxin production were reported to be necessary to maintain the redox balance in ticks (Kusakisako et al., 2016; Della Noce et al., 2019). It has been proposed that tick microbiome would be influenced by oxidative stress (Narasimhan and Fikrig, 2015), and different AMP would have different inhibitory consequence on microbial growth (Saito et al., 2009; Zhang et al., 2015). These immune responses in ticks may work together and eventually influence the viral and bacterial composition of a specific host blood meal through a complicated pathway.

On the other hand, the redox situation may indirectly affect pathogen transmission by changing its balance with other microflora in the ticks as suggested in mosquitoes (Cirimotich et al., 2011). We noticed that the gene encoding reeler domain-containing gut protein was significantly upregulated in the fully fed ticks (Supplementary Table 3). A protein of *Ixodes scapularis* with a Reeler domain (PIXR) was induced upon feeding and upregulated in *Borrelia burgdorferi*-infected *I. scapularis* tick guts. PIXR might influence the tick gut microbiome composition by regulating the ability of gram-positive bacteria to form biofilms in the gut when the tick takes a blood meal (Narasimhan et al., 2017). These alterations may influence *B. burgdorferi* entering the tick gut in multiple ways (Zhang et al., 2015). We identified that the gene encoding a protein similar to PIXR was upregulated in the fully fed *I. persulcatus* ticks; however, its role in the transmission of *Borrelia* and other tick-borne pathogens deserves further study.

We noted several limitations as follows: The interpretation of differential gene expression between the flat and fully fed ticks might be over-cautious because we used pooled samples with a relatively limited sample size. Using laboratory-reared ticks from a single colony can decrease the bias caused by individual tick differences; however, this study examined bloodmeal-induced gene regulation and virome in ticks originating from the field and feeding on human patients. We thereby gained valuable information on the risks to patients from tick-borne viruses, at the expense of likely uniformity in response to blood-feeding amongst the studied ticks. We suggest that further research with more field and laboratory-derived samples, and experiments from proteomics and functional genomics perspectives, could be performed to verify our findings.

## Data Availability Statement

The datasets presented in this study can be found in online repositories. The names of the repository/repositories and accession number(s) can be found below: https://www.ncbi.nlm.nih.gov/, PRJNA639641.

## Ethics Statement

The studies involving human participants were reviewed and approved by Mudanjiang Forest Central Hospital 2011-03. The patients/participants provided their written informed consent to participate in this study.

## Author Contributions

All authors contributed to the manuscript and approved the submitted version.

## Conflict of Interest

The authors declare that the research was conducted in the absence of any commercial or financial relationships that could be construed as a potential conflict of interest.

## Publisher’s Note

All claims expressed in this article are solely those of the authors and do not necessarily represent those of their affiliated organizations, or those of the publisher, the editors and the reviewers. Any product that may be evaluated in this article, or claim that may be made by its manufacturer, is not guaranteed or endorsed by the publisher.

## Supplementary Material

Supplementary Figure 1 RNAseq analysis of tick gene transcripts detected in pools of flat and fully fed *Ixodes persulcatus* ticks removed from human patients at Mudanjiang Forestry Central Hospital in Heilongjiang Province, China. Heatmap with clusters showing the Pearson correlation matrix for pairwise sample comparisons of differentially expressed genes in flat (pools TG1, TG2, and TG13), and fully fed (pools TG5, TG6, and TG15) ticks.

Supplementary Figure 2 RNAseq comparison of tick gene transcripts detected in pools of flat and fully fed *I. persulcatus* ticks removed from human patients at Mudanjiang Forestry Central Hospital in Heilongjiang Province, China. Taxonomic classification of differentially expressed genes with similar expression patterns based on the Clusters of Orthologous Groups (COG) database (A) COG classification of genes upregulated in fed versus unfed ticks; (B) COG classification of genes downregulated in fed versus unfed ticks.
