# Supplementary material for: Virome and Blood Meal-Associated Host Responses in Ixodes persulcatus Naturally Fed on Patients
Source: Front Microbiol. 2022 Feb 17;12:728996. doi: 10.3389/fmicb.2021.728996 (PMC8891964; doi:10.3389/fmicb.2021.728996)
Supplement: Supplementary file 1 [file Table_1.DOCX]

**Table S1. RNAseq analysis of pools of *Ixodes persulcatus* ticks removed from human patients at Mudanjiang Forestry Central Hospital in Heilongjiang Province, China. Identification of reads corresponding to host cox1 gene sequences in nine libraries derived from pools of ten flat (TG1, TG2, TG13), partially-fed (TG3, TG4, TG14) and fully-fed (TG5, TG6 and TG16) ticks.**

| **Blastx_hit** | Identity | PValue | E-value |
| --- | --- | --- | --- |
| **Up-regulated** |  |  |  |
| XP_002410252.1 secreted salivary gland peptide, putative [*Ixodes scapularis*] | 75 | 1.30E-08 | 52 |
| XP_002403996.1 cystatin, putative [*Ixodes scapularis*] | 89.4 | 0.000303 | 66 |
| XP_002402814.1 coiled-coil domain-containing protein, putative [*Ixodes scapularis*] | 76.5 | 0.000622 | 34 |
| XP_002403134.1 secreted glycine rich protein, putative [*Ixodes scapularis*] | 80.8 | 5.29E-05 | 78 |
| AGQ57038.1 vitellogenin receptor [*Amblyomma hebraeum*] | 47.8 | 0.000658 | 69 |
| CAL51265.1 anticomplement protein IxAC-B4 precursor [*Ixodes ricinus*] | 72.9 | 0.000679 | 85 |
| XP_002435043.1 chitinase, putative [*Ixodes scapularis*] | 90.7 | 0.000683 | 118 |
| XP_002402114.1 secreted protein, putative [*Ixodes scapularis*] | 83.3 | 0.000795 | 48 |
| XP_002400094.1 conserved hypothetical protein [*Ixodes scapularis*] | 75 | 4.41E-08 | 96 |
| ABL61513.1 Der-p2-like allergen [*Ixodes ricinus*] | 56.6 | 1.96E-05 | 145 |
| **Down-regulated** |  |  |  |
| XP_002411805.1 hypothetical protein IscW_ISCW021794 [*Ixodes scapularis*] | 58.7 | 6.21E-11 | 63 |
| AAT92216.1 ATP synthase c-subunit [*Ixodes pacificus*] | 100 | 2.53E-14 | 152 |
| XP_002411805.1 hypothetical protein IscW_ISCW021794 [*Ixodes scapularis*] | 60 | 7.63E-15 | 65 |
| XP_002409611.1 cuticular protein, putative [*Ixodes scapularis*] | 97 | 0.000405 | 100 |
| AAT92170.1 ribosomal protein L6 [*Ixodes pacificus*] | 100 | 2.53E-07 | 68 |
| XP_002414860.1 apoptosis-promoting RNA-binding protein TIA-1/TIAR, putative [*Ixodes scapularis*] | 100 | 2.78E-09 | 113 |
| XP_002415051.1 4SNc-Tudor domain protein, putative [*Ixodes scapularis*] | 94.2 | 1.09E-12 | 69 |
| XP_002403093.1 hypothetical protein IscW_ISCW000986 [*Ixodes scapularis*] | 90.9 | 4.86E-13 | 121 |
| AAM93590.1 putative secreted protein [*Ixodes scapularis*] | 76.8 | 2.19E-19 | 69 |
| XP_002407601.1 valacyclovir hydrolase, putative [*Ixodes scapularis*] | 98.1 | 0.000672 | 322 |

**Table S2. RNAseq analysis of tick gene transcripts detected in pools of flat and fully-fed *Ixodes persulcatus* ticks removed from human patients at Mudanjiang Forestry Central Hospital in Heilongjiang Province, China.** **Blastx results for the top 10 genes differentially-expressed between**

**flat and fully-fed ticks.**

**Table S3. RNAseq analysis of tick gene transcripts detected in pools of flat and fully-fed *Ixodes persulcatus* ticks removed from human patients at Mudanjiang Forestry Central Hospital in Heilongjiang Province, China. The logFC differences in expression of certain important genes are shown as positive numbers for down-regulated genes and negative numbers for up-regulated**

**genes.**

| Gene_name | logFC | *P* Value |
| --- | --- | --- |
| **Antioxidant genes** |  |  |
| peroxidase | 2.82156506 | 0.000641846 |
| glutathione peroxidase | 4.306895503 | 0.000125303 |
| protein kinase C [contig 1] | 3.783932368 | 1.22E-09 |
| protein kinase C [contig 2] | 2.501821803 | 6.17E-07 |
| glutathione S-transferase [contig 1] | 2.865224037 | 7.39E-06 |
| glutathione S-transferase [contig 2] | 2.785822712 | 0.000343914 |
| glutathione S-transferase [contig 3] | 2.683648817 | 1.67E-05 |
| peroxiredoxin | 2.82156506 | 0.000641846 |
| **Antimicrobial peptide** |  |  |
| lysozyme | 2.228577205 | 0.000384005 |
| **Reeler domain-containing gut protein [*Ixodes scapularis*]** | -2.589157504 | 4.05E-07 |
