# Supplementary material for: Virome and Blood Meal-Associated Host Responses in Ixodes persulcatus Naturally Fed on Patients
Source: Front Microbiol. 2022 Feb 17;12:728996. doi: 10.3389/fmicb.2021.728996 (PMC8891964; doi:10.3389/fmicb.2021.728996)
Supplement: Supplementary Figure 1 — RNAseq analysis of tick gene transcripts detected in pools of flat and fully fed Ixodes persulcatus ticks removed from human patients at Mudanjiang Forestry Central Hospital in Heilongjiang Province, China. Heatmap with clusters showing the Pearson correlation matrix for pairwise sample comparisons of differentially expressed genes in flat (pools TG1, TG2, and TG13), and fully fed (pools TG5, TG6, and TG15) ticks. [file Image_1.PDF]

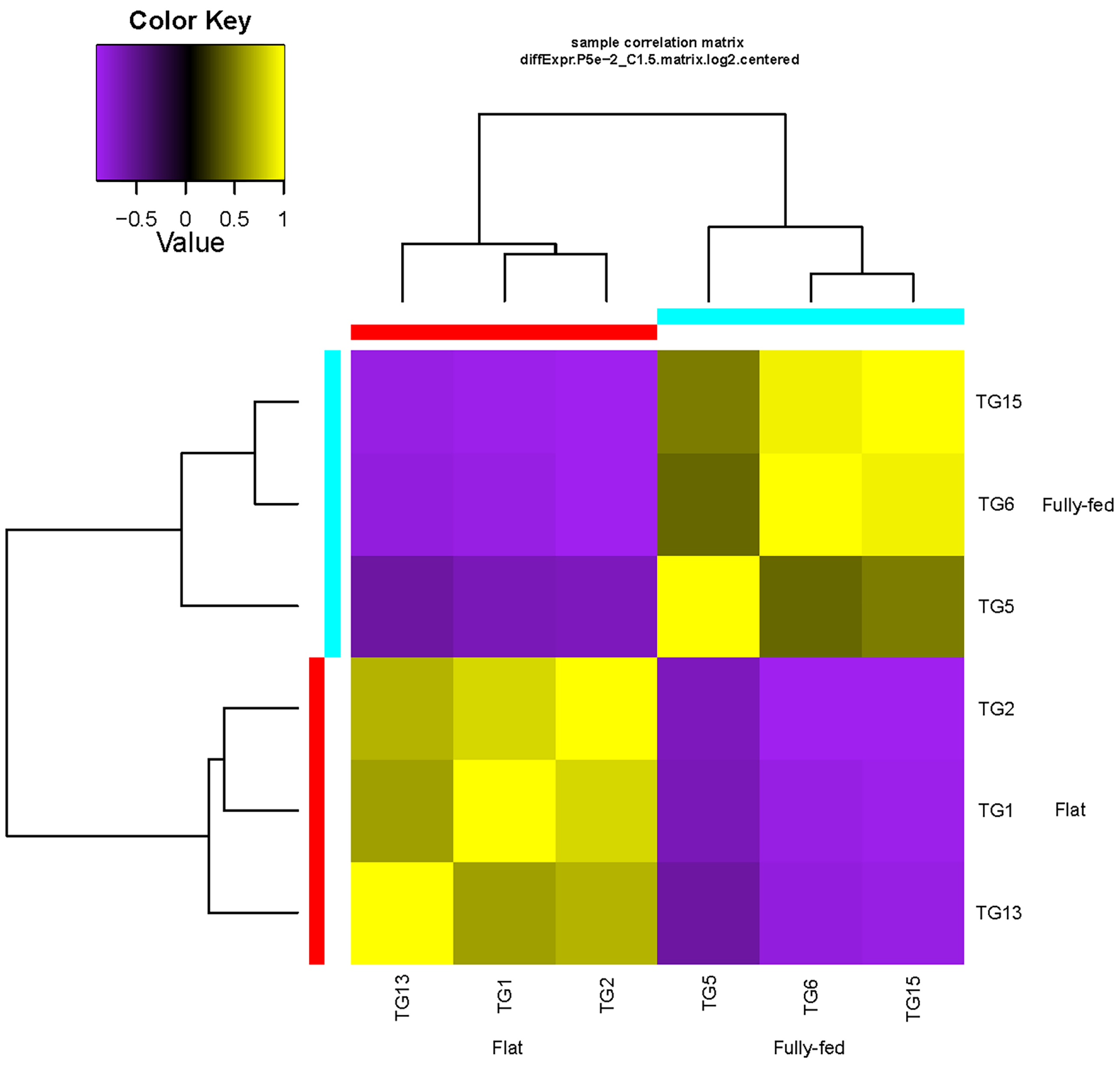
Color Key
−0.5 0 0.5 1
Value
sample correlation matrix
diffExpr.P5e−2_C1.5.matrix.log2.centered
TG15
TG6
Fully-fed
TG5
TG2
TG1
Flat
TG13
TG13
TG1
TG2
TG5
TG6
TG15
Flat
Fully-fed

**Figure S1.** RNAseq analysis of tick gene transcripts detected in pools of flat and fully-fed *Ixodes persulcatus* ticks removed from human patients at Mudanjiang Forestry Central Hospital in Heilongjiang Province, China. Heatmap with clusters showing the Pearson correlation matrix for pairwise sample comparisons of differentially-expressed genes in flat (pools TG1, TG2, and TG13) and fully-fed (pools TG5, TG6, and TG15) ticks.

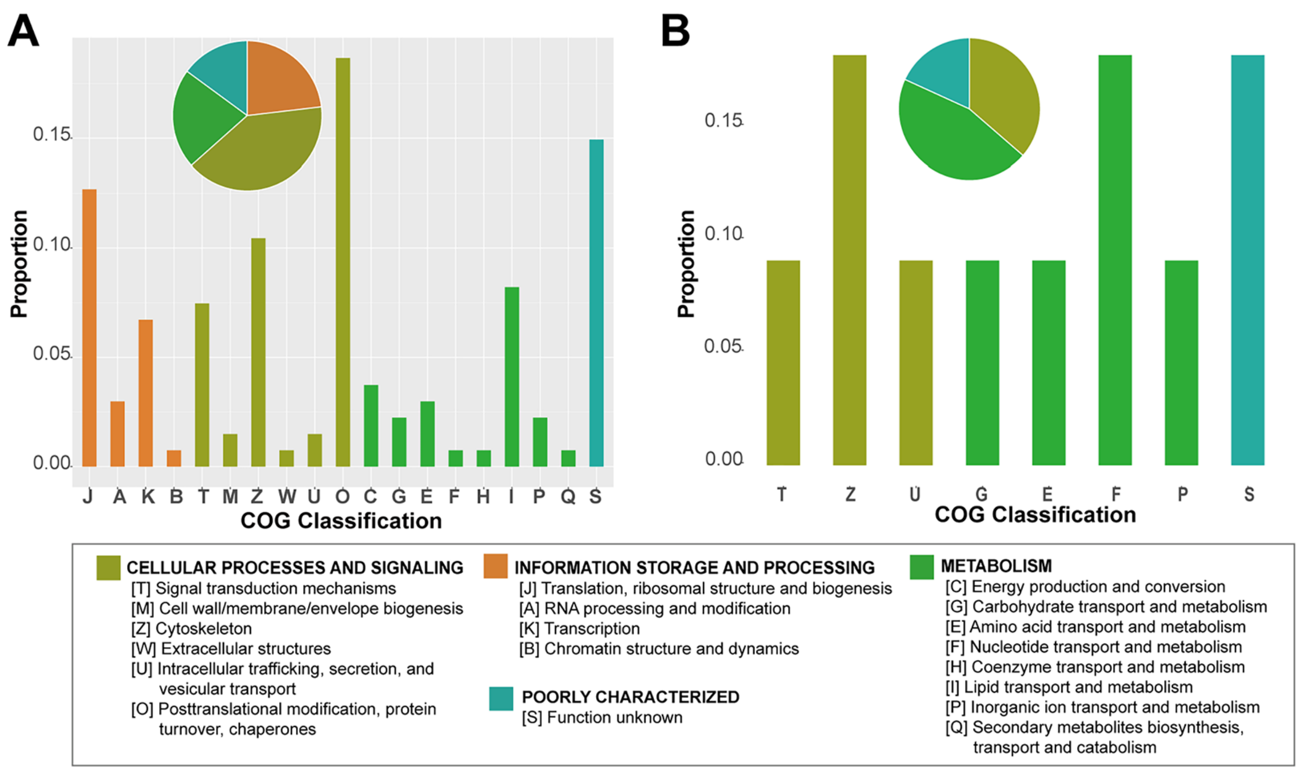
A
B
Proportion
0.15
0.10
0.05
0.00
J A K B T M Z W U O C G E F H I P Q S
T Z U G E F P S
COG Classification
CELLULAR PROCESSES AND SIGNALING
[T] Signal transduction mechanisms
[M] Cell wall/membrane/envelope biogenesis
[Z] Cytoskeleton
[W] Extracellular structures
[U] Intracellular trafficking, secretion, and vesicular transport
[O] Posttranslational modification, protein turnover, chaperones
INFORMATION STORAGE AND PROCESSING
[J] Translation, ribosomal structure and biogenesis
[A] RNA processing and modification
[K] Transcription
[B] Chromatin structure and dynamics
POORLY CHARACTERIZED
[S] Function unknown
METABOLISM
[C] Energy production and conversion
[G] Carbohydrate transport and metabolism
[E] Amino acid transport and metabolism
[F] Nucleotide transport and metabolism
[H] Coenzyme transport and metabolism
[I] Lipid transport and metabolism
[P] Inorganic ion transport and metabolism
[Q] Secondary metabolites biosynthesis, transport and catabolism

**Figure S2.** RNAseq comparison of tick gene transcripts detected in pools of flat and fully-fed *Ixodes persulcatus* ticks removed from human patients at Mudanjiang Forestry Central Hospital in Heilongjiang Province, China. Taxonomic classification of differentially-expressed genes with similar expression patterns based on the Clusters of Orthologous Groups (COG) database (A) COG classification of genes up-regulated in fed versus unfed ticks; (B) COG classification of genes down-regulated in fed versus unfed ticks.
